# Advances in spiral fMRI: A high-resolution dataset

**DOI:** 10.1016/j.dib.2022.108050

**Published:** 2022-03-12

**Authors:** Lars Kasper, Maria Engel, Jakob Heinzle, Matthias Mueller-Schrader, Nadine N. Graedel, Jonas Reber, Thomas Schmid, Christoph Barmet, Bertram J. Wilm, Klaas Enno Stephan, Klaas P. Pruessmann

**Affiliations:** a Institute for Biomedical Engineering, ETH Zurich and University of Zurich, Gloriastrasse 35, Zurich 8092, Switzerland; b Translational Neuromodeling Unit, Institute for Biomedical Engineering, University of Zurich and ETH Zurich, Wilfriedstrasse 6, Zurich 8032 Switzerland; c Wellcome Centre for Integrative Neuroimaging, FMRIB, Nuffield Department of Clinical Neurosciences, University of Oxford, Oxford, United Kingdom; d Wellcome Centre for Human Neuroimaging, University College London, London WC1N 3BG, United Kingdom; e Max Planck Institute for Metabolism Research, Cologne 50931, Germany

**Keywords:** ISMRMRD, MR image reconstruction raw data, Magnetic field monitoring, Non-cartesian MRI, Task-based fMRI, High-resolution fMRI, Ultra high-field spiral MRI, Inverse problems

## Abstract

We present data collected for the research article “Advances in Spiral fMRI: A High-resolution Study with Single-shot Acquisition” (Kasper et al. 2022). All data was acquired on a 7T ultra-high field MR system (Philips Achieva), equipped with a concurrent magnetic field monitoring setup based on 16 NMR probes. For task-based fMRI, a visual quarterfield stimulation paradigm was employed, alongside physiological monitoring via peripheral recordings.

This data collection contains different datasets pertaining to different purposes:

(1) Measured magnetic field dynamics (k_0_, spiral k-space trajectories, 2nd order spherical harmonics, concomitant fields) during ultra-high field fMRI sessions from six subjects, as well as concurrent temperature curves of the gradient coil, to explore MR system and subject-induced variability of field fluctuations and assess the impact of potential correction methods.

(2) MR Raw Data, i.e., coil and concurrent encoding magnetic field (trajectory) data, of a single subject, as well as nominal spiral gradient waveforms, precomputed B_0_ and coil sensitivity maps, to enable testing of alternative image reconstruction approaches for spiral fMRI data.

(3) Reconstructed image time series of the same subject alongside behavioral and physiological logfiles, to reproduce the fMRI preprocessing and analysis, as well as figures presented in the research article related to this article, using the published analysis code repository.

All data is provided in standardized formats for the respective research area. In particular, ISMRMRD (HDF5) is used to store raw coil data and spiral trajectories, as well as measured field dynamics. Likewise, the NIfTI format is used for all imaging data (anatomical MRI and spiral fMRI, B_0_ and coil sensitivity maps).

## Specifications Table

**Table:** 

Subject	*Neuroimaging*
Specific subject area	*Functional Magnetic Resonance Imaging; Ultra-high field strength (7 Tesla) MRI; Model-based image reconstruction; Magnetic Field Monitoring; Inverse Problems*
Type of data	Magnetic Resonance (MR) Images Raw MR Coil Data and K-Space Trajectory Data Magnetic Field Monitoring Data Temperature Sensor Curves Physiological Monitoring Traces Behavioral Logfiles
How the data were acquired	MR Data: 7T MRI, Philips Achieva; quadrature transmit/32-channel head receive array coil (Nova Medical); fMRI data with spiral trajectories Concurrent Magnetic Field Monitoring: Custom NMR field probe setup (16 fluorine-based NMR field probes on a laser-sintered nylon frame [2]; Acquisition with dedicated NMR spectrometer [3]; Processing [4] on separate computer using Matlab R2018a Gradient Coil Temperature: Custom setup with 5 optical sensors cast into the gradient coil [5], custom acquisition software on separate computer Visual Stimulation: VisuaStim LCD goggles (Resonance Technology Inc., Northridge, CA, USA) using Matlab R2017a and Cogent 2000 (v1.32) Physiological Monitoring: Philips MR standard peripheral recordings via respiratory bellows and finger pulse plethysmograph
Data format	Raw and filtered
Description of data collection	Data was acquired from seven healthy volunteers (4 female, mean age 25.7 +/- 4.1 y), who were instructed to lie still in the scanner while fixating a central target during task-based fMRI (block design visual quarterfield stimulation). One subject (SPIFI_0001) was excluded from further analysis due to hardware-related insufficient data quality (reduced signal in receiver coil channels).
Data source location	Institution: ETH Zurich (Swiss Federal Institute of Technology) City/Town/Region: Zurich Country: Switzerland Latitude: 47.376855 Longitude: 8.550307
Data accessibility	Repository name: *ETH Research Collection (hosted by the Library of the Swiss Federal Institute of Technology, Zurich, CH)* Data identification number: https://www.research-collection.ethz.ch/handle/20.500.11850/487412 Direct URL to data: https://doi.org/10.3929/ethz-b-000487412 Instructions for accessing these data: Unrestricted download from ETH research collection (file-by-file), licensed under CC-BY 4.0 Share-Alike
Related research article	L. Kasper, M. Engel, J. Heinzle, M. Mueller-Schrader, N.N. Graedel, J. Reber, T. Schmid, C. Barmet, B.J. Wilm, K.E. Stephan, K.P. Pruessmann, Advances in spiral fMRI: A high-resolution study with single-shot acquisition, NeuroImage. 246 (2022) 118738. https://doi.org/10.1016/j.neuroimage.2021.118738.

## Value of the Data

• This data is valuable to the scientific community, because it comprises a public collection of raw magnetic resonance (MR) data for non-Cartesian (spiral) image acquisition at ultra-high field (7 Tesla) in the context of functional MR imaging (fMRI). Such raw data (coil data and encoding k-space trajectory before image reconstruction) is required to study image reconstruction, artifact formation and sensitivity of MRI. Other existing raw data repositories, e.g., NYU fastMRI [6], focus on structural, Cartesian imaging at lower field strength.
• MR researchers who are interested in advancing non-Cartesian image reconstruction can benefit from this data, because it provides input and calibration (B0/sensitivity map) files in standardized data formats (ISMRMRD, NIfTI) that allow usage in major MR image reconstruction packages (e.g., BART, MIRT, Gadgetron, MRIReco.jl [7], [8], [9], [10]). As our optimized image reconstruction data is also included, comparative studies can be performed based on this data collection.
• The inclusion of concurrent magnetic field monitoring data from 6 different imaging sessions allows to study the variability in magnetic field dynamics at ultra-high field during fMRI, including system-dependent changes in the magnetic field, as well as physiologically induced field fluctuations. Such data is also useful for simulating the characteristics and magnitude of field-induced image artifacts, to evaluate potential correction mechanisms (e.g., navigator echoes, gradient impulse response function measurements).
• The reconstructed single-subject time series (NIfTI) constitutes ultra-high field fMRI data at sub-millimeter resolution, and is well controled due to its simple visual stimulation task. Because the point-spread function of the employed spiral imaging differs from typical rectilinear echo-planar imaging (EPI), it provides complementary information to study the spatial specificity of the BOLD response, e.g., to inform layer-specific fMRI analyses.
• Both magnitude and phase images are provided for the fMRI time series. The conjugate gradient SENSE-based coil combination provides phase images with high signal-to-noise ratio (SNR), while field monitoring removes background field fluctuations. Therefore, the data is suited to study the functional sensitivity of phase fMRI at ultra-high field.

## 1 Data Description

### 1.1 Data for studying field dynamics

We introduce the data in this collection by different categories pertaining to their intended use (Table 1). For studying field dynamics (data category 1), and to assess the impact of potential correction methods, we provide magnetic field monitoring data of six subjects (``SPIFI_000[2–7]_FieldDynamics_spiralOut.h5''), stored in hierarchical data format (HDF5) with the ISMRM raw data (ISMRMRD) specification [11]. The measured field dynamics are stored in the ``traj'' field of the ISMRMD format and represent time-courses of spatial basis set coefficients, consisting of a global phase term $k_{0}$, the k-space trajectory $k_{x},k_{y},k_{z}$, as well as 2nd order spatial spherical harmonics ($xy,\;zy,\;3z^{2}-r^{2},\;xz,\;x^{2}-y^{2}$). Furthermore, quadratic concomitant field terms are stored, computed from the monitored k-space trajectory [12]. The reference coordinate system of the basis functions is the scanner geometry, given by the x,y,z gradient field directions (vendor convention of Philips: +x: anterior, +y: left, +z: foot for supine head-first positioning of the subject, with the origin in magnet iso-center).

**Table 1:** List of files in this data collection. ``SPIFI_000[2-7]'' as part of the file name indicates the subject identifier of the corresponding file. Category numbers: (1) Data for Studying Field Dynamics; (2) Data for Image Reconstruction; (3) Data for fMRI Processing, Analysis and Figure Reproduction.

Category	File	Description
1	GradientCoilTemperature_AllSubjects.zip	Gradient coil temperature curves (.CSV) during whole MRI scanning session for all subjects, accompanied by a README.md markdown file with a more detailed data description
1	SPIFI_000 [2–7]_FieldDynamics_spiralOut.h5	Measured magnetic field dynamics of each subject during high-resolution spiral fMRI run, up to 2nd spatial order spherical harmonics, including separate concomitant fields (scanner coordinate system, monitored for every 3rd of 36 slices, volumes 001-100, ISMRMRD file)
2	SPIFI_0007_MapsForReconstruction.zip	Processed B_0_ and coil sensitivity maps used for reconstruction, raw B_0_ map (NIfTI files)
2	SPIFI_0007_RawData_RotatedToSliceGeometry3D_spiralOut_monitored_singleVolume050.h5	Single volume raw data (ISMRMRD) for high-resolution spiral, coil signal and monitored trajectory (rotated to 2D reconstruction plane, 36 slices, volume 050)
2	SPIFI_0007_RawData_RotatedToSliceGeometry3D_spiralOut_monitored_volumes[001-033|034-066|067-100].h5	Complete fMRI run raw data (ISMRMRD, 3 parts) for high-resolution spiral; coil signal and monitored trajectory (rotated to 2D reconstruction plane, 36 slices, volumes 001-033/034-066/067-100)
2	SPIFI_0007_RawData_ScannerGeometryHigherOrderFields_spiralOut_monitored_singleVolume050.h5	Subject SPIFI_0007: Single volume raw data (ISMRMRD) for high-resolution spiral; coil signal and monitored field dynamics (up to 2nd spatial order, separate concomitant fields, scanner coordinate system, 36 slices, volume 050)
1, 2	SPIFI_0007_RawData_ScannerGeometryHigherOrderFields_spiralOut_nominal_singleVolume050.h5	Subject SPIFI_0007: Single volume raw data (ISMRMRD) for high-resolution spiral; coil signal and nominal trajectory (separate concomitant fields, scanner coordinate system, 36 slices, volume 050)
3	SPIFI_0007_ReconstructedImagesAndLogfiles.zip	Subject SPIFI_0007: All reconstructed images and logfiles to reproduce fMRI analysis (NIfTI and ASCII text files)

Each file contains the magnetic field dynamics acquired during an ultra-high resolution (0.8 mm) spiral-out fMRI run at 7 Tesla for every 3rd of 36 imaging slices (TR 270 ms) and all volumes. Each time-course starts about 1 ms before the spiral gradient waveform onset and covers its whole duration, with a sampling rate of 1 MHz. No coil data is provided in these files, and the corresponding ``data'' field therefore contains a single vector of zeros for each field dynamics time-course.

In addition, to relate changes in the MR system to the observed field fluctuations, we provide temperature curves of the gradient coil during the entire imaging session of each subject (``GradientCoilTemperature_AllSubjects.zip''), as measured by 5 optical sensors cast into the gradient coil [5]. The curves are stored in column-based ASCII files (CSV format), with each row representing a temperature readout every 18–19 s. The absolute temperature (in degree Celsius) is not accurate (offset of about +18 degrees Celsius), but the temperature differences are to scale. We indicate the start of the spiral fMRI runs in an additional column (accuracy of about 10 s). More details and other time stamp codes can be found in the README.md file within the zip archive.

### 1.2 Raw data for image reconstruction

For a single subject (SPIFI_0007), we provide all raw data, as well as calibration maps for image reconstruction, of the ultra-high resolution spiral fMRI data (data category 2). This includes ISMRMRD files containing raw coil data from the 32 receive channels, as well as first order k-space coefficients. Compared to the field dynamics data above (category 1), the trajectory data here is rotated to the patient coordinate system, defined by the slice geometry, i.e., current imaging plane (indicated by keyword “RotatedToSliceGeometry3D” in the file name). A transformation between both coordinate systems is possible via the rotation matrix defined by the "read_dir, phase_dir, slice_dir'' vectors in the ISMRMRD header. The data field contains the demodulated coil data, removing phase effects of the measured global field and orthogonal gradients (see Methods, Raw Data Processing), to enable direct gridding-based image reconstruction without further processing. Time-courses in both ``traj'' and ``data'' field are cropped to the duration of the spiral gradient waveform. Because of file size restrictions (10 GB), the raw data of the full fMRI run (36 slices, 100 volumes) is split into 3 parts (``SPIFI_0007_RawData_RotatedToSliceGeometry3D_spiralOut_monitored_volumes [001-033|034-066|067-100].h5'').

For quick test reconstructions, the 50th (central) volume of this dataset has also been exported separately (``SPIFI_0007_RawData_RotatedToSliceGeometry3D_spiralOut_monitored_singleVolume050.h5''). To enable higher-order reconstructions and facilitate the conversion between coordinate systems when merging data of category 1 and 2, the raw coil data (without demodulation) of this volume is also provided with the phase coefficients in the scanner coordinate system, including the 2nd order spatial spherical harmonics, and equal cropping. (``SPIFI_0007_RawData_ScannerGeometryHigherOrderFields_spiralOut_monitored_singleVolume050.h5''). Finally, the same coil data is paired with the nominal trajectory represented in the scanner coordinate system, computed from the target spiral gradient waveform supplied to the MR system, to enable studying the fidelity of gradient waveform execution and necessity of calibration or correction methods (e.g., provided by the field monitoring data above). (``SPIFI_0007_RawData_ScannerGeometryHigherOrderFields_spiralOut_nominal_singleVolume050.h5'').

Alongside the raw spiral data, preprocessed maps (smoothed and resized) of B_0_ (in Hz) and coil sensitivity (split into magnitude and phase) are provided as image files (NIfTI, ``SPIFI_0007_MapsForReconstruction.zip''). These maps are required as calibration data for the spiral image reconstruction. The raw B_0_ map (``b0MapRaw1_Hz.nii'') is included for completeness, such that alternative processing methods can be explored.

### 1.3 Data for fMRI processing, analysis and figure reproduction

All data required for fMRI analysis is contained in a single zip-archive (``SPIFI_0007_ReconstructedImagesAndLogfiles.zip''), pertaining to the same subject (SPIFI_0007) as the raw data presented in the previous section. The subfolder scandata contains all image files (NIfTI format), i.e., the reconstructed images of the ultra-high resolution spiral out fMRI time series (``fmri1.nii''), enabling comparison to other reconstruction methods, as well as the multi-echo anatomical reference data (``me1.nii''), which can be used to re-create the calibration maps for image reconstruction. It further contains the spiral-in/out fMRI run with separate files for the reconstruction of the in and out-part of the readout, as well as their SNR-optimal image combination (``fmri2_[In|Out|Combined]*.nii''). In addition to the magnitude data, phase images are provided for each image set (file suffix ``_phase'').

Behavioral logfiles of both runs (``fmri[1|2]_behav.[log|mat]'') are located in the behavior subfolder, and contain visual paradigm time stamps as well as subject's button responses and TTL trigger events from the scanner. The Matlab file was generated by the custom paradigm script and contains meta-information on the paradigm settings, while the column-based ASCII text file is the standard output log of the Matlab Cogent Toolbox used for stimulus presentation.

Physiological logfiles of both runs (``fmri[1|2]_phys.log'') are located in the physlog subfolder, and contain respiratory and cardiac traces, as well as scanner timing information via gradient waveform samples. The file format is a vendor-specific (Philips) column-based ASCII file, with new values appended as new rows every 2 ms (500 Hz sampling rate).

## 2 Experimental Design, Materials and Methods

### 2.1 Setup and subjects

All data were acquired on a Philips Achieva 7 Tesla MR System (Philips Healthcare, Best, The Netherlands), with a quadrature transmit coil and 32-channel head receive array (Nova Medical, Wilmington, MA).

Spiral trajectory and magnetic field dynamics were measured concurrently via 16 fluorine-based NMR field probes positioned between transmit and receive head coil elements, on a laser-sintered nylon frame [2]. A dedicated NMR spectrometer acquired and preprocessed (filtering, demodulation) the probe data with 1 MHz bandwidth [3] before phase extraction and spherical harmonic basis set expansion [4] on a separate computer using Matlab R2018a.

Gradient coil temperature curves were acquired for each scan session, sampling relative temperature difference (in degree Celsius) every 18-19 s from 5 optical sensors cast into the gradient coil [5]. Custom acquisition software on the fMRI stimulus computer recorded these readouts, allowing synchronization via system clock and logged TTL triggers. With this information, onset time stamps for each fMRI run were added as an additional column to the raw CSV logfiles. Because of trigger delays, wait periods and the sampling rate of the temperature measurements, this timing is accurate only up to about 10 s.

Visual stimulation for task-based fMRI was performed by VisuaStim LCD goggles (Resonance Technology Inc., Northridge, CA, USA), using Matlab R2017a and Cogent 2000 (v1.32).

Peripheral physiological recordings of respiratory and cardiac traces were acquired during each fMRI run with a 500 Hz sampling rate, using the MR system's standard respiratory bellows and finger pulse plethysmograph.

Data was acquired from seven healthy volunteers (4 female, mean age 25.7 +/- 4.1 y), who were instructed to lie still in the scanner while performing the experimental task. One subject (SPIFI_0001) was excluded from further analysis due to hardware-related insufficient data quality (reduced signal in receiver coil channels).

### 2.2 fMRI Paradigm

Subjects underwent a block-design visual stimulation experiment, where two alternating blocks of visual quarter-field presentation (15 s duration each) were interleaved with equally long fixation periods.

Visual quarter-field stimulation was induced by flickering checkerboard wedges: In one block, upper left and lower right visual field were stimulated simultaneously (condition ULLR). In the other block, checkerboard wedges flickered in the upper right and lower left quarter-fields (condition URLL). To ensure subjects’ attention, they were asked to report slight contrast changes in the central fixation target via button presses of the right hand. The paradigm was programmed in Matlab R2017a, using Cogent 2000 (v1.32), and synchronized to the imaging sequence via a TTL trigger signal from the scanner fed into the serial port of the stimulus computer.

For the ultra-high resolution spiral fMRI data presented here, as well as the spiral in/out image time series, a single 5 min run of the paradigm was performed per subject (5 repetitions of the ULLR-Fixation-URLL-Fixation sequence).

### 2.3 Image acquisition

Spiral Sequence Parameters:

For the functional MRI data, 2D ultra-high resolution (0.8 mm, FOV 230 mm) single-shot spiral readouts were employed, with 4-fold radial undersampling, a total readout time of 57 ms, echo time (TE) 20 ms and a preceding 10 ms SPIR fat suppression module [13]. 36 slices (0.9 mm thickness, 0.1 mm gap) were acquired in an ascending slice order with a volume TR of 3.3 s. Slice geometry was chosen oblique-transverse to cover the visual cortex, with an RL-axis rotation aligning slices parallel to the calcarine sulcus. The whole fMRI run contained 100 volume repetitions, i.e., a total duration of 5.5 min. A second functional run was performed using a combined spiral in/out readout (1.5 mm resolution) with equal slice geometry, FOV and undersampling, but shorter total readout duration of 39 ms and slightly longer TE of 25 ms.

Anatomical Scan and Calibration Maps:

A multi-echo Cartesian spin-warp gradient echo scan (TR 800 ms, $TE_{1}$ 4 ms, ΔTE 1 ms) with 1 mm in-plane resolution was acquired, serving as anatomical reference and source data to compute the calibration maps (B_0_ maps and receive coil sensitivity maps). Slice geometry was matched to the spiral sequence.

Magnetic Field Monitoring:

Concurrent magnetic field monitoring at 1 MHz bandwidth was performed with the 16 NMR probes for the spiral gradient waveform of every 3rd slice (TR 270 ms) and all volumes of the fMRI runs. The monitoring window started about 1 ms before the spiral gradient waveform and lasted for the whole readout of about 57 ms.

### 2.4 Raw data processing

Magnetic Field Monitoring Data (traj):

Magnetic field dynamics were computed via a basis set expansion [4], projecting the unwrapped phase time courses of the field probes onto 2nd order spatial spherical harmonics basis functions [14], i.e., the global term $k_{0}$, the k-space trajectory $k_{x},k_{y},k_{z}$, as well as 2nd order spatial spherical harmonics ($xy,\;zy,\;3z^{2}-r^{2},\;xz,\;x^{2}-y^{2}$). Quadratic concomitant fields were computed analytically from the monitored k-space trajectory [12]. Field monitoring data was smoothed with a raised cosine filter (50 kHz bandwidth), synchronized to the head coil data, and resampled to its dwell time (1.8 µs).

Afterwards, two processing pipelines were employed, depending on the usage of the data for studying field dynamics (category 1) or image reconstruction (category 2). For studying field dynamics, data was kept uncropped, retaining about 1 ms of data before the onset of the spiral readout, and remained in the coordinate system given by the x,y,z gradient field directions. All readouts of one run were saved in a single ISMRMRD file [11] using the ISMRMD Matlab tools and the script ``save_k_ismrmrd.m'' within our custom analysis code repository [15].

The second processing pipeline catering to image reconstruction cropped the field monitoring data to include only the samples acquired during the spiral readout. Furthermore, it allowed to store the data either in the scanner coordinate system (file keyword ``ScannerGeometry'') or rotate it to the patient coordinate system (file keyword ``RotatedToSliceGeometry3D''), defined by the slice geometry, i.e., the current imaging plane, which facilitate its direct use in 2D gridding-based image reconstruction. In this case, higher order field terms were discarded and the field dynamics were saved alongside the processed coil data as ISMRMRD files (script ``save_rawdata_ismrmrd.m'' in our GitHub analysis repository [15]).

Coil data (data):

The raw data, sampled with a dwell time of 1.8 µs, was cropped to include only the samples acquired during the spiral readout. If combined with field dynamics in the scanner coordinate system (file keyword “ScannerGeometry”), no further processing ensued.

If used for gridding-based image reconstruction (files labeled “RotatedToSliceGeometry”), coil data was demodulated (multiplied by exp(−iφ(t)) with two different phase terms φ(t), induced by the monitored global field ($\varphi \left(t\right)=k_{0}\left(t\right)$) and by the gradients orthogonal to the reconstruction plane ($\varphi \left(t\right)=k\left(t\right)\cdot r_{0}$), with $r_{0}$ being the respective slice offset [2].

Then, the processed coil data was saved together with the processed field monitoring data (pipeline 2), as described above, using ``save_rawdata_ismrmrd.m''.

### 2.5 Image reconstruction

Image reconstruction of the ultra-high resolution spiral fMRI data was performed using an iterative conjugate gradient (cg-)SENSE algorithm [16], incorporating an expanded signal model of the imaging process [14], i.e., the first-order monitored k-space trajectory, as well as calibration maps for recovering undersampled data (sensitivity maps) and correction of static magnetic field inhomogeneity (B_0_ maps). Reconstruction speed was accelerated via k-space gridding of the 2D spiral trajectory (represented in the patient coordinate system, i.e., “RotatedToSliceGeometry“) and Fast Fourier Transform [16], as well as multi-frequency interpolation (MFI) for B_0_ off-resonance correction [17]. 10 iterations of the cg-SENSE algorithm were performed to reconstruct each slice, using an in-house Matlab code implementation (Matlab R2018a). Reconstruction was parallelized over slices and volumes using the high-performance computing cluster of ETH Zurich. All reconstructed images were finally saved as 4D NIfTI files using SPM12.

The calibration maps itself were computed before the spiral reconstruction, based on images of the multi-echo Cartesian reference scan reconstructed with the same algorithm (but omitting B_0_ correction). From the first echo image, complex-valued sensitivity maps were computed by normalized single coil reconstructions, i.e., pixel-wise division by the root sum of squares of all channel images. Raw B_0_ maps were calculated from the phase images of all echoes by pixel-wise linear regression. B_0_ and sensitivity maps were smoothed via a variational approach [18], and interpolated to the targeted image reconstruction size of the spiral data.

Example reconstruction code in Julia [19] for the ultra-high resolution spiral fMRI raw data (Data Description, category 2) is provided in a GitHub repository [20] (https://github.com/mrikasper/julia-recon-advances-in-spiral-fmri). This code utilizes the cg-SENSE implementation provided by MRIReco.jl [9], which includes static B_0_ map correction via an alternative approximation [21] to the MFI used above.

### 2.6 Reproduction of fMRI analysis pipeline

To reproduce the fMRI preprocessing, analysis, and figures for the research article [1] corresponding to this data collection, the files in category 3 (see Table 1) can be used.

Data analysis and visualization was performed in Matlab R2019b, based on SPM 12 (Wellcome Centre for Human Neuroimaging, London, UK, http://www.fil.ion.ucl.ac.uk/spm/), as well as the UniQC [22] and PhysIO [23] Toolboxes, whose open-source code is available as part of the TAPAS software collection: https://www.translationalneuromodeling.org/tapas [24].

All custom Matlab code is bundled in a GitHub repository [15] (https://github.com/mrikasper/paper-advances-in-spiral-fmri), with submodules referencing the external packages.

There are separate main scripts for reproducing the analysis pipeline (``main.m''), and the visualization of the manuscript figure components (``main_create_figures.m''). Note that only the single subject results for SPIFI_0007 can be recreated, and path settings might have to be adapted in the code (see the ``README.md'' in the repository for details).

## Ethics Statements

Data was obtained from healthy human volunteers after written informed consent, and with approval of the local ethics committee (Swissmedic 10000216).

## CRediT authorship contribution statement

**Lars Kasper:** Conceptualization, Methodology, Software, Formal analysis, Investigation, Resources, Data curation, Writing – original draft, Writing – review & editing, Project administration. **Maria Engel:** Conceptualization, Methodology, Software, Investigation, Resources, Writing – review & editing. **Jakob Heinzle:** Conceptualization, Methodology, Writing – review & editing. **Matthias Mueller-Schrader:** Methodology, Software, Formal analysis, Validation. **Nadine N. Graedel:** Methodology, Formal analysis, Validation, Writing – review & editing. **Jonas Reber:** Methodology, Resources. **Thomas Schmid:** Methodology, Resources. **Christoph Barmet:** Conceptualization, Methodology, Resources. **Bertram J. Wilm:** Methodology, Software, Resources. **Klaas Enno Stephan:** Conceptualization, Resources, Writing – review & editing, Funding acquisition. **Klaas P. Pruessmann:** Conceptualization, Methodology, Resources, Writing – original draft, Supervision, Funding acquisition.

## Declaration of Competing Interest

At the time of submission, Christoph Barmet and Bertram J. Wilm are employees of Skope Magnetic Resonance Technologies. Klaas P. Pruessmann holds a research agreement with and receives research support from Philips. He is a shareholder of Gyrotools LLC. Rest authors have no conflicts of interest to declare.
